# Reducing disease burden through sustainable diets: a modeling approach for national food-based dietary guidelines

**DOI:** 10.3389/fnut.2026.1826425

**Published:** 2026-07-17

**Authors:** Lisa Sturm, Lena Klausmann, Katrin Seper-Nagl, Oliver Alber, Antonia Griesbacher, Tilman Kühn, Klemens Fuchs, Karl-Heinz Wagner, Alexandra Wolf

**Affiliations:** 1 Division Integrative Risk Assessment, Data and Statistics, Department Center for Nutrition and Prevention, Austrian Agency for Health and Food Safety, Vienna, Austria; 2 Vienna Doctoral School of Pharmaceutical, Nutritional and Sport Sciences (PhaNuSpo), Vienna, Austria; 3 Division Integrative Risk Assessment, Data and Statistics, Department Statistics and Analytical Epidemiology, Austrian Agency for Health and Food Safety, Vienna, Austria; 4 Department of Nutritional Sciences, University of Vienna, Vienna, Austria; 5 Department of Nutritional, Food and Consumer Sciences, Fulda University of Applied Sciences, Fulda, Germany; 6 Center for Public Health, Medical University of Vienna, Vienna, Austria; 7 Co-Center for Sustainable Food Systems and Institute for Global Food Security, Queen’s University Belfast, Belfast, United Kingdom; 8 Division Integrative Risk Assessment, Data and Statistics, Austrian Agency for Health and Food Safety, Vienna, Austria

**Keywords:** food-based dietary guidelines, healthy diets, mathematical optimization, planetary health, sustainable diets

## Abstract

**Introduction:**

Shifting to sustainable dietary patterns is essential for improving human and planetary health. Current dietary habits substantially contribute to non-communicable diseases and environmental impacts, particularly due to high consumption of animal-based foods. Food-based dietary guidelines (FBDGs) that integrate nutritional, health, and environmental dimensions are therefore increasingly important. While global frameworks highlight the need for sustainable diets, national guidelines must reflect cultural and regional contexts to ensure feasibility and acceptance. Mathematical optimization has emerged as a promising approach to develop such guidelines.

**Methods:**

This study aimed to update the Austrian FBDGs using mathematical optimization, incorporating nutritional adequacy, burden of disease expressed as disability-adjusted life years (DALYs), cultural acceptability, greenhouse gas emissions (GHGE), and land use (LU). Linear programming was applied to identify dietary patterns that meet nutritional requirements, minimize environmental and health impacts, and remain close to observed Austrian dietary intake. The optimized model outputs formed the scientific foundation for translating results into consumer-oriented national dietary guidelines.

**Results:**

The optimized diet met all nutrient recommendations and macronutrient distribution ranges. The resulting FBDGs emphasize higher intakes of plant-based foods, particularly vegetables, fruits, grains, and legumes, alongside substantially lower amounts of animal-based products. Meat intake should not exceed 300 g per week, while legumes are recommended at least three times per week. Compared with the current Austrian diet, the optimized dietary pattern suggests a potential reduction in diet-related health burden and environmental impacts (DALYs -43%, GHGE -58%, LU -61%), highlighting its potential to improve public health and reduce environmental pressures.

**Discussion:**

The updated Austrian FBDGs provide clear, quantitative recommendations that simultaneously ensure nutritional adequacy, reduce disease burden, and support environmental sustainability. As current meat consumption substantially exceeds recommendations, gradual dietary shifts are essential. Increasing legumes and minimally processed legume-based products can facilitate this transition. Effective implementation will require broad communication efforts and supportive food environments to the enable adoption of healthier and more sustainable dietary patterns.

## 1 Introduction

For human and planetary health, shifting to more sustainable dietary patterns is essential to ensure that by 2050 healthy diets are accessible to everyone worldwide (1). Globally, suboptimal diets are attributable to a total of 11 million deaths (2) and 255 million disability-adjusted life-years (DALYs) (3). In addition, around 70% ($8,1 trillion) of the hidden costs of the agrifood systems are related to unhealthy diets and are linked to non-communicable diseases (NCDs) (4). Furthermore, food systems are responsible for a third of global greenhouse gas emissions (GHGE) (5). Especially animal-based foods, such as red meat, tend to have a greater environmental footprint than plant-based foods (6). Therefore, the implementation of healthy food-based dietary guidelines (FBDGs) that include environmental considerations in addition to nutrient adequacy and chronic disease prevention is a major milestone to achieve the Sustainable Development Goal 2 (Zero hunger, SDG2) (1). While the EAT-Lancet Commission (7, 8) has proposed a global framework for a Planetary Health Diet to optimize both individual and planetary health, several countries (9, 10) have recently updated their FBDGs taking into the account cultural acceptability of dietary recommendations and specific public health needs on a national level. There are various methods used for deriving FBDGs. For example, the European Food Safety Authority (EFSA) has defined seven steps for developing FBDGs (11). As FBDGs should not only be designed to meet nutritional requirements and to facilitate prevention but also consider the environmental impact of current food systems and local food preferences, mathematical optimization is a promising new approach to integrate these domains (12). Mathematical diet optimization is also recommended by the FAO (13). The objective of our study was to update the current Austrian FBDGs using mathematical optimization, considering nutritional adequacy, burden of disease, cultural acceptability as well as essential environmental indicators.

## 2 Materials and methods

This study presents a constrained optimization model for developing sustainable and healthy food-based dietary guidelines, based on the German Nutrition Society’s (DGE) model (14), which has been adapted and further developed for Austria. The model consists of decision variables, constraints, and an objective function. Linear programming is used to determine optimal food quantities that meet nutritional, environmental, and health-related goals in agreement with national dietary preferences.

### 2.1 Optimization process and objectives

#### 2.1.1 Input data

In the present study, nationally representative dietary intake data from the Austrian Nutrition Report 2017 (multistage stratified cluster sampling design) (15) from 2,129 Austrian adults aged 18–64 years (63% women, 37% men) (16, 17), were used (hereinafter referred to as “observed diet”). Foods were classified using the FoodEx2 system at level 4, resulting in 547 food groups (decision variables), later aggregated into 20 main food groups for reporting of results and to 9 FBDG-food groups. In addition to the 20 main food groups, so called discretionary foods were reported as the summary of their energy share (Supplementary 1).

Data on the nutritional content of foods was obtained from German Food Code and Nutrient Data Base (18) and the Austrian Food Composition Database (19). Data on environmental indicators –GHGE and land use (LU) – were sourced from the SHARP indicator database (20). Health impact data, expressed in DALYs, were derived from the Global Burden of Disease Study (3) and of Schwingshackl et al. (21).

#### 2.1.2 Model constraints

In order to prevent the models from generating unrealistic intake values, acceptability constraints were applied. The maximum possible intake for each food group was set at the 95th percentile of the observed intake for all individuals for each food group at level 1 and additionally, for each food group on level 4, the 95th percentile for consumers only. A different approach was employed based on Schäfer et al. (22), for “composite dishes” and “seasoning and sauces,” which are included in the discretionary food groups. Additionally, based on the average amount consumed in the observed diet, a daily limit of 80 mL was established for fruit juices. Additional constraints were also applied on total quantity of food, total solid quantity of food and total energy intake. The equations of the constraints are presented in Supplementary 2.

#### 2.1.3 Objective function, nutrient goals, agronomic dependencies and DALY calculation

As already described by Sturm et al. (23), the optimization aimed to minimize the:

- Deviation from observed intake.
- Environmental impact.
- Burden of disease (DALYs).

The optimization problem and the objective function f can be expressed as


$$\text{minimiz}e_{x^{\mathrm{opt}}}\;f=[\begin{array}{c} W^{\mathrm{DA}}\times \text{deviation food habits}+\\ W^{\mathrm{SUS}}\times \text{environmental impact}+\\ W^{\mathrm{DHR}}\times \text{health impact} \end{array}]+P\times \text{dfnr}$$

$$\text{with}\;W^{\mathrm{DA}}+W^{\mathrm{SUS}}+W^{\mathrm{DHR}}=1,$$

$$\text{deviation food habits}=\frac{\sum_{i=1}^{n}\|(x_{i}^{\mathrm{opt}}-x_{i}^{\mathrm{obs}})\|}{\text{Total}\;\mathrm{obs}\;\text{amount}\;}\;\text{or}\;\frac{\sum_{i=1}^{n}\frac{\left\|(x_{i}^{\mathrm{opt}}-x_{i}^{\mathrm{obs}})\right\|}{x_{i}^{\mathrm{obs}}}}{n},$$

$$\text{environmental impact}=(\sum_{i=1}^{n}\alpha \;(\frac{x_{i}^{\mathrm{opt}}\times \mathrm{GHG}E_{i}}{\text{Total}\;\mathrm{obs}\;\text{GHGE}})+\beta \;(\frac{x_{i}^{\mathrm{opt}}\times LU_{i}}{\text{Total}\;\mathrm{obs}\;\mathrm{LU}})),$$

health impact=∑i=1nδ×xiopt×DALYGBDiTotalobsDALYsGBD+∂×xiopt×DALY_SCHWiTotalobsDALYs_SCHWand

$$\begin{array}{c} \text{dfnr}=\sum_{k=1}^{\begin{array}{c} k=\text{nutrients}\\ \min \end{array}}\mathrm{De}v_{k}^{-}+\sum_{l=1}^{\begin{array}{c} l=\text{nutrients}\\ \max \end{array}}\mathrm{De}v_{l}^{+}\\ +\sum_{m=1}^{\begin{array}{c} m=\text{nutrients}\\ \text{target} \end{array}}\frac{\left\|(\mathrm{nu}t_{m}^{\mathrm{opt}}-\mathrm{DR}V_{m}^{\text{target}})\right\|}{\mathrm{DR}V_{m}^{\text{target}}}\;\text{with} \end{array}$$

$$\mathrm{De}v_{l}^{+}=\{\begin{array}{c} \frac{\mathrm{nu}t_{l}^{\mathrm{opt}}-\mathrm{DR}V_{l}^{\max}}{\mathrm{DR}V_{l}^{\max}}\;\text{if}\;\mathrm{nu}t_{l}^{\mathrm{opt}}\geq \mathrm{DR}V_{k}^{\max}\\ 0\;\text{if}\;\mathrm{nu}t_{l}^{\mathrm{opt}}<\mathrm{DR}V_{k}^{\max} \end{array}$$

$$\text{subject to}\;g_{t},t=1,\ldots ,5$$

where W^DA^, W^SUS^, and W^DHR^ are weights controlling dimensions, *n* is the number of selected food groups, $x_{i}^{\mathrm{opt}}$ and $x_{i}^{\mathrm{obs}}$ are the optimized and selected amounts of food group i respectively, GHGE_i_ and Lu_i_ represent carbon impact and land use expressed per gram of selected food group i , and the weights *α* and *β* control the influence of the environmental metrics GHGE and land use (LU). *Total obs GHGE* and *total obs LU* are the total amounts of GHGE and LU in the observed diet. ${\text{DALY}}_{\mathrm{GBDj}}\;\text{and}\;\mathrm{DAL}Y_{\text{SCHW}j}$ , respectively, stand for the diet health relationship metrics of the Global Burden of Disease Study (GBD) (3) and Schwingshackl et al. (21), expressed per gram of the selected food group j . The weights 𝛿 and 𝜕 control the health influence of the health relationship metrics. Total obs $\text{DALY}s_{\mathrm{GBD}}\;$ and total obs $\text{DALY}s_{\text{Schw}}$ are the total amounts of GBD and Schwingshackl metrics of the observed diet. P is the nutritional deviation tolerance weight. $\mathrm{De}v_{k}^{-}$ is inadequate intake (negative deviations) and $\mathrm{De}v_{l}^{+}\;$ is the excess (positive deviations). k , l , and m are numbers of nutrients having a minimum, maximum and target goal, respectively. $\;\mathrm{nu}t_{k}^{\mathrm{opt}}$ is the optimized amount of nutrient k . $\mathrm{DR}V_{k}^{\min}\;$ is the minimum DRV (dietary reference value) associated with nutrient j . $\mathrm{DR}V_{l}^{\max}$ is the maximum DRV associated with nutrient l , and $\mathrm{DR}V_{m}^{\text{target}}$ is the exact target value to reach for nutrient m.

The additional component *dfnr* (*deviation from nutritional requirements*) aims to minimize deviations from minimal nutritional values to reach, to minimize excess for nutrients having a maximal value, and to minimize deviations from nutritional targets. P was assigned a high value to effectively penalize the objective function value if the solution deviates from nutrient goals.

The terms $g_{t}(t=1,\ldots 5)$ refer to particular inequality and equality constraints for each selected food group, food group level, total quantity, total solid quantity, and total energy intake, respectively.

Additionally, nutrient goals had to be met. The dietary reference values (DRVs) for Austria (24) and the EFSA’s tolerable upper intake levels for nutrients (25), respectively, served as the foundation for the nutrient goals (Supplementary 3). DRVs for adults of 18–65 years of age, normal weight and with a moderate level of physical activity (PAL 1.4) were used and weighted according to the age and sex-specific proportion of the Austrian population. Due to limited data quality in the nutritional databases used, some nutrients (iodine, selenium, copper, fluoride and manganese) had to be excluded (18, 19). Since dietary intake alone is insufficient to reach the DRV of Vitamin D, no lower limit was established (26). Agronomic dependencies for milk to butter (100:2, 5–5 g) (27), milk to beef (100:2 kg (28)) as well as red unprocessed meat to processed meat [in the range of 100:80–120 g (29)] were considered as a nutrient in the objective function.

The DALYs for the optimized diet were calculated using the following approach:


$$\text{DALYs}\;\mathrm{per}\;100\;g\;\text{of food group}\;i=\frac{\left(\text{DALY}s_{i}^{\mathrm{Obs}}-0\;\text{DALYs}\right)}{\left(x_{i}^{\mathrm{Obs}}-x_{i}^{\text{TMREL}}\right)}\times 100$$

$$\text{DALY}s_{\mathrm{opt}}=\sum_{x_{i}}\frac{\text{DALYs}\;\mathrm{per}\;100\;g\;\text{of}\;i\times x_{i}^{\mathrm{opt}}}{100}$$

The assumptions on which the development of this diet-health indicator was based have been explained in full in other places (30).

The optimization was implemented with the R programming language and for practical use, particularly for testing different values in the constraints or additional intake data, an interactive web application in R shiny was made available.

### 2.2 Sensitivity analyses

Different weightings were tested for the three components $W^{\mathrm{DA}}$ , $W^{\mathrm{SUS}}$ , and $W^{\mathrm{DHR}}$ of the objective function – deviation from observed dietary intake, environmental impact and health impact – in order to evaluate the sensitivity and robustness of the model results. Within their component of the objective function, DALYs from GBD and Schwingshackl, as well as GHGE and LU, were given equal weighting. Our analyses indicated that a stronger consideration of the environmental and health components produced comparable optimization outcomes. In contrast, increasing the weight assigned to ‘deviation from observed dietary intake’ led to more pronounced deviations from the optimized pattern. To systematically evaluate this effect, the weighting assigned to the ‘deviation from observed dietary intake’ parameter was varied in increments of five across the interval [0, 100], while maintaining equal weighting between environmental and health components. A stepwise approach was used to ensure that resulting dietary patterns remained practical and realistic. The current study also aimed to reduce the environmental impact of the resulting dietary patterns by at least 45% because reducing GHGE by this amount by 2030 could limit global warming to 1.5 °C with a 50% probability (31, 32). Based on these considerations, the final weighting scheme assigned 40% to ‘deviation from observed dietary intake’, and 30% each to ‘environmental impact’ and ‘health impact’.

### 2.3 Derivation of omnivorous FBDGs for Austria

The dietary pattern resulting from optimization (scenario 1), without additional constraints to those described in chapter 2.1.2, led to a dietary pattern with very limited amounts of meat, but very high amounts of fish (Supplementary 4). Scenario 1 was reviewed and discussed with a wide range of national and international experts, as well as over 40 stakeholders from the National Nutrition Commission of the Federal Ministry of Social Affairs, Health, Care and Consumer Protection (33). The commission comprises of various and heterogeneous stakeholders such as members from the Federal Ministry of Labour, Social Affairs, Health, Care and Consumer Protection, as well as other relevant ministries, representatives from the federal states, professional societies, universities, institutions, social partners and social insurance providers.

During stakeholder meetings the projected meat intake of 11 g per day (equivalent to < 1 serving per week) was discussed as difficult to implement, as the current meat consumption of the Austrian population is 10 times higher (15). The majority of stakeholders agreed that such a drastic reduction would not be accepted by large parts of the population. A gradual reduction in meat consumption was considered more advisable. In addition, agricultural conditions in Austria, such as the high proportion of grassland (51%), were considered as relevant for the dietary recommendations (34). Similarly, the calculated fish intake of 52 g per day (equivalent to around 2.5 weekly servings) was considered to be difficult to achieve among the Austrian population and would require additional promotion, given that fish is not consumed to this extent in Austria (15). Due to the lack of access to the sea, Austria’s self-sufficiency rate for fish is currently only 7% (35).

Therefore, 3 other scenarios were developed (see chapter 2.3.1).

#### 2.3.1 Final modeling scenarios

In total, 4 types of optimization scenarios were developed for discussion in the National Nutrition Commission and their working groups (Table 1). In scenario 1 only the constraints in chapter 2.1.2 were applied. Scenario 2 contains an additional constraint for 43 g of meat per day based on the Planetary Health Diet (7) and recommendations of the WCRF (36) (equals max. 300 g of meat per week, 2 portions of meat per week). Compared to scenario 1, scenario 3 contains an additional constraint for 38 g of fish per day (corresponds to 266 g fish per week, or 2 portions of fish per week) based on the observed diet. Compared to scenario 1, Scenario 4 contains an additional constraint for 32.25 g meat per day (equals 226 g meat per week or 1.5 portions of meat per week).

**Table 1:** Description of the different scenarios.

Scenarios	Constraints
Observed diet	–
Scenario 1	As described in chapter 2.1.2
Scenario 2	As described in chapter 2.1.2 + 2 portions of meat per week
Scenario 3	As described in chapter 2.1.2 + 2 portions of fish per week
Scenario 4	As described in chapter 2.1.2 + 1.5 portions of meat per week

#### 2.3.2 Adjustment of the modeling results

The results of the different scenarios (Supplementary 4) were reviewed and discussed in an iterative process over several meetings with the National Nutrition Commission of the Federal Ministry of Social Affairs, Health, Care and Consumer Protection. Scenario 4 was chosen as the basis for the derivation of FBDGs. To better reflect the consumer reality and the regional conditions in Austria the food groups “fish and seafood,” “eggs” and “milk and dairy products” were slightly adjusted thereafter, resulting in a dietary pattern with 1.5 servings of meat and 1.5 servings of fish per week (adjusted scenario 4) (Supplementary 4). A control model of the adjusted scenario 4 was run, to ensure that the chosen diet fulfills all goals set.

The final results were then converted into recommended daily or weekly portions of practical serving sizes to provide consumers with practical information.

## 3 Optimization results

The nutrient contribution of the observed and optimized diet (adapted scenario 4) is shown in Supplementary 5. For the content of main food groups (g) of the observed diet and the optimized diet see Table 2.

**Table 2:** Content of main food groups (g) of the observed diet and the optimized diet.

Food groups and subgroups	Observed diet (g/d)	Optimized diet (g/d)
Drinking water	1,626	1,039
Coffee and tea	532	126
Vegetables and fruit	394	672
Vegetables	183	331
Fruit	131	261
Fruit and vegetable juices	80	80
Grains, grain-based products and potatoes	301	378
Refined grains (−products)	229	247
Whole grains (−products)	17	17
Potatoes	55	114
Legumes and legume products	14	37
Legumes (cooked)	12	13
Plant-based meat substitutes made of legumes	2	24
Eggs	23	26
Milk equivalents*	571	411
Milk/dairy (in milk equivalents)	561	361
Plant-based drinks	9	50
Fats, oils, nuts and seeds	25	26
Vegetable oils	8	10
Nuts and seeds	8	8
Spreadable fats	9	8
Meat	147	32
Red meat	56	6
Processed meat	44	5
Poultry	28	21
Fish and seafood	19	29
Discretionary foods	21 E%	10 E%

^*^A milk equivalent is a measure used to calculate the amount of milk used in a dairy product. Conversion of dairy products into milk equivalents (71): Milk factor 1; Dairy products factor 1.4; Cheese factor 7.2.

### 3.1 Observed (current) diet

The observed diet does not meet several nutritional requirements. Specifically, intake levels of total fat, saturated fat, cholesterol, dietary fibre, free sugars, folate, calcium, iron, potassium and sodium deviated from recommended targets. Total energy intake of the observed diet is 2,250 kcal, with macronutrient distribution comprising 50E% (percentage of daily energy intake) carbohydrates, 37 E% fat and 78 g protein per day. Refined grains account for the largest share of energy intake (30 E%), followed by milk and dairy products (13 E%). Overall, animal-based foods contribute 30 E% to total energy intake. In the observed diet, 44% of total protein comes from plant-based foods and 56% from animal-based products. The primary contributors to total protein are grains (36%), milk and dairy products (20%) and red meat (14%).

The environmental impact of the observed diet is quantified as 5.9 kg CO₂-equivalents (GHGE) and 7.3 m^2^ (LU) per person per day (Table 3). Animal-based foods are responsible for the majority of GHGE (61%), with red meat alone contributing 23%. Red meat also represents the largest share of land use impact (32%).

**Table 3:** Overview of GHGE, LU, and DALYs of the observed and optimized diet.

Environmental and health indicators	Observed diet	Optimized diet
GHGE (kg CO_2_-eq/day)	5.86	2.8
LU (m^2^/year/day)	7.34	3.5
Reduction in DALYs	–	43%

### 3.2 Optimized diet

In comparison to the observed diet, the optimized diet is within the acceptable distribution ranges for macronutrients and meets all established nutrient goals. It provides a total energy intake of 2029 kcal/day, with macronutrient contributions of 53% E% carbohydrates, 30 E% fat, and a total of 64 g of protein per day. Animal-based foods contribute 19 E% to the total energy intake in the optimized diet. In contrast to the observed diet, plant-based foods account for 65% of total protein intake. The primary protein sources include grains (33%), followed by milk and dairy products (15%), and legumes and legume products (9%). Compared to the observed diet, the optimized diet features increased intake of vegetables, fruits, legumes, and plant-based meat alternatives, alongside a substantially reduced intake of meat. Compared to the observed diet, the intake of discretionary foods (in E%) in the optimized diet is halved.

Limiting nutrients and therefore the strongest determinants of the dietary changes required from the observed diet were calcium, zinc, iron and Vitamin B_12_. Calcium intake in the optimized diet (1,000 mg/d) was slightly higher than in the observed diet (959 mg/d). In the optimized diet, milk and dairy products were the main source of calcium (45% of total amount of calcium), followed by vegetables (17% of total amount of calcium), especially green leafy vegetables such as spinach. Zinc content shall be 11 mg/d in the optimized diet and 12 mg/d in the observed diet. Refined grains (34% of total amount of zinc), milk and dairy products (18% of total amount of zinc) and vegetables (12% of total amount of zinc) contribute most of the zinc supply. Vitamin B_12_ content in the optimized diet (4 μg/d) is lower than in the observed diet (5.5 μg/d) but still meets the DRVs. The main sources are fish and seafood (35% of total B_12_), milk and dairy products (26% of total amount of B_12_), eggs (10% of total amount of B_12_) and processed meat (8% of total amount of B_12_). Iron content shall be 15 mg/d in the optimized diet and 12 mg/d in the observed diet, respectively. Refined grains (30% of total amount of iron) and vegetables (29% of total amount of iron) were the predominant sources.

The environmental impact of the optimized diet was quantified as 2.8 kg CO₂-equivalents (GHGE) and 3.5 m^2^ (LU) per person per day (Table 2). Animal-based foods made up the majority of GHGE (52%), with 20% of total GHGE from milk and dairy products. Milk and dairy products also represented the largest share of LU impact (16%). The optimized diet would result in a potential reduction of 43% DALYs compared to the observed diet-related burden of disease in the Austrian population (Table 3).

### 3.3 The new Austrian food pyramid for an omnivorous diet

The results from dietary optimization modeling were used to formulate FBDGs for omnivorous individuals. Quantitative outputs, expressed in grams per day, were translated into practical recommendations for daily or weekly consumption using standardized portion sizes (Table 4). For example, one portion of egg was defined as 60 g; thus, an optimized intake of 26 g/day corresponds to approximately 182 g per week, or three eggs weekly. The resulting FBDGs (37) motivate a relatively plant-based dietary pattern with smaller amounts of meat and fish.

**Table 4:** National omnivorous FBDGs of Austria.

Food group	Recommended daily or weekly portions	Portion size
Non-alcoholic beverages	6 portions/day	250 mL
Vegetables and fruit	5 portions/day	Vegetables, cooked: 200–300 g Vegetables, raw: 100–200 g Salad: 75–100 g Fruit: 125–150 g
Grains and potatoes	4 portions/day	Bread: 50–70 g Rolls: 50–70 g Cereals: 50–60 g Pasta, uncooked: 65–80 g Rice, cooked: 150–180 g Potatoes: 200–250 g
Legumes and legume products	at least 3 portions/week	Legumes, cooked: 125 g Tofu, Tempeh etc.: 80 g
Milk and dairy	2 portions/day	Milk: 150–200 mL Yoghurt: 150–200 g Cheese: 50–60 g
Eggs	3 portions/week	60 g (1 egg)
Meat and fish	1 portion of meat/week + 1 portion of fish/week + 1 extra portion of meat or fish per week	Meat: 150 g Fish: 130–150 g
Vegetable oils, nuts and seeds	2 portions/day	Vegetable oil: 1 tablespoon (10 g) Nuts and seeds: 2 tablespoons (25 g)
Foods high in fat, sugar and salt	Occasionally	

#### 3.3.1 The pyramid base: daily food choices

The base of the pyramid is composed of non-alcoholic beverages with a recommended minimum daily intake of 1.5 liters. Preference is given to water or other non-alcoholic, low-calorie beverages such as unsweetened fruit or herbal teas. Black tea or coffee in moderation (up to four cups per day in total) can be part of a healthy diet.

The next tier includes vegetables and fruits, which are good sources of vitamins, minerals, and secondary plant metabolites (18). A daily intake of five portions is recommended, ideally distributed as three portions of vegetables and two portions of fruit. A practical guide suggests that one clenched fist approximates one portion. Choosing seasonal and locally grown varieties supports both nutritional variety and environmental sustainability.

Grains and potatoes provide complex carbohydrates, fiber, vitamins, and minerals (18). Four portions per day are advised, with an emphasis on whole grain options. Portion sizes are defined as one palm-sized piece for bread and two fist-sized servings for cooked starchy foods such as potatoes, pasta, or rice.

Milk and dairy products supply calcium, Vitamin B_12_, protein, iodine, and Vitamin B_2_ (18). Two daily portions are recommended, favoring low-fat and unsweetened options. Ideally, one portion should consist of “white” dairy products (e.g., milk, yogurt, buttermilk, cottage cheese) and one of “yellow” dairy products (e.g., cheese). One portion equals a glass/cup of 150–200 mL or two thin slices (palm-sized) for solid varieties.

Plant-based drinks are not nutritionally equivalent to cow’s milk due to differences in nutrient composition (18). If milk and dairy intake is below the recommendation, unsweetened plant-based alternatives (e.g., soy drinks) with added calcium, Vitamin B_12_, and Vitamin B_2_ should be chosen.

Daily intake of plant-based oils, nuts and seeds is recommended due to their content of essential fatty acids. Two portions per day are advised. One portion of oil equals one tablespoon. One portion of seeds/nuts equals approximately two tablespoons. Spreadable fats, cooking fats such as butter, margarine, or lard, and high-fat dairy products like cream, sour cream or crème fraîche should be used sparingly. Cooking methods that minimize fat use, such as steaming, boiling, or grilling, are preferable to deep frying. Oils rich in omega-3 fatty acids are particularly recommended.

#### 3.3.2 The middle tier: weekly food choices

The updated food pyramid emphasizes plant-based protein sources, including legumes and minimally processed meat alternatives such as tofu, tempeh, and textured soy protein. Legumes (lentils, beans, chickpeas, peas, soybeans, lupins) provide protein, fiber and micronutrients (18) and should be consumed at least three times a week. One portion of cooked legumes is approximately 125 g (one clenched fist). One portion of tofu or tempeh equals 80 g (a palm-sized, finger-thick piece).

Marine fish provides iodine and fatty marine fish offers valuable omega-3 fatty acids (DHA and EPA) (18). Preference should be given to fish from sustainable fisheries or responsible aquaculture. Local freshwater fish such as trout and carp offer comparable omega-3 content to lean marine fish and represent viable regional alternatives. If no marine fish is consumed, an additional tablespoon of rapeseed oil per day is advised to compensate for omega-3 intake. One portion of fish á 130–150 g per week is recommended (a palm-sized, finger-thick piece). Meat contains protein, iron, Vitamin B_12_, selenium and zinc (18). If included in the diet, intake should be limited to one portion of lean meat á 150 g (palm-sized piece) per week. Red meat (e.g., beef, pork, lamb) and processed meat should be consumed less frequently. Optionally, an additional portion of fish or meat (in total max. 300 g per week) may be consumed weekly.

Up to three eggs per week are recommended, contributing Vitamin B_12_ and protein (18).

#### 3.3.3 The apex of the pyramid: foods for occasional consumption

Foods high in fat, sugar, and salt (HFSS), such as confectionery, baked goods, savory snacks, and sugar-sweetened beverages, should be consumed only occasionally and in small quantities. A portion is defined as one small handful.

#### 3.3.4 Additional considerations

Complementary to the graphical illustration as a food pyramid, additional guidance is provided on current topics such as the consumption of processed foods, mindful cooking and eating practices, reduction of food waste, and the promotion of regular physical activity (37). These elements support a holistic approach to sustainable and health-promoting dietary behavior.

## 4 Discussion

The present paper describes the process and results of updating the Austrian omnivorous FBDGs using a comprehensive and modern approach, which was absolutely novel for Austria. To transform the food system and support the SDGs, more countries must commit to creating FBDGs that specifically highlight the critical connection between the local diet and planetary health (1). In 2022, James-Martin et al. (38) concluded that only about 17% of the global population is covered by FBDGs that address environmental sustainability. Since then, several countries in Europe have updated their FBDGs to incorporate environmental indicators (9, 10, 14, 39–43). Mathematical optimization has been successfully applied in the development of sustainable FBDGs in countries such as the Netherlands, Germany, and Switzerland (9, 14, 39). Although the methodological approaches differ, the resulting dietary recommendations demonstrate a high degree of consistency across countries. When compared to other recent dietary recommendations such as the Planetary Health Diet, Netherlands FBDGs, Germanys FBDGs, Norways FBDGs, the Nordic Nutrition Recommendations (NNR) and the UK’s Eatwell Guide, several key similarities but also differences emerge that highlight regional and cultural differences. While the Planetary Health Diet focuses on a global diet for healthy individuals aged 2 years and older (7, 8), other recommendations are mostly aimed at the general population of each country, region or specific target groups like children (9, 10, 14, 44). However, the Planetary Health Diet was not intended to be a rigid dietary recommendation. Instead, it was designed as a framework for guidance of national FBDGs worldwide (7, 8).

### 4.1 Differences in specific food groups of the Austrian FBDGs compared to other countries

#### 4.1.1 Legumes

Legumes are key plant-based protein sources, rich in fiber and essential minerals like iron and zinc (45, 46). Environmentally, legumes have low climate impacts and improve soil health by fixing nitrogen and adapting well to dry, nutrient-poor conditions (47, 48). Combining legumes with grains ensures a complete amino acid profile, as their protein compositions complement each other (49–51). Although legumes currently contribute only 0.2% to Austria’s protein intake, consumption has tripled since 2006, indicating a growing trend (52), however still very low. Recent FBDGs increasingly emphasize the importance of legumes as part of a shift toward more plant-based diets (protein transition). Previously grouped with vegetables, they therefore form a distinct category in the updated Austrian FBDGs, highlighting their nutritional and environmental value.

The Austrian FBDGs recommend three servings per week, corresponding to approximately 37 g/day in an omnivorous diet. In the vegetarian version of the Austrian guidelines, legumes play an even more central role (23, 37). The Planetary Health Diet similarly highlights legumes, recommending an intake of approximately 50 g/day, with a flexible range of 0–100 g/day (7). In contrast, the recommendations from Germany and the Netherlands are significantly lower (9, 14). The Nordic Nutrition Recommendations (NNRs) and recommendations of Norway do not specify exact amounts but emphasize that legumes should be a significant part of the diet (10, 43). For example, Norway recommends consuming legumes at least once per week (43). The UK Eatwell Guide includes legumes within the broader “beans, pulses, fish, eggs, meat” group, encouraging their consumption but without providing quantitative targets (44). Austria’s inclusion of legumes as a weekly staple aligns with broader international trends and reinforces their role in sustainable and health-promoting diets. Notably, the Austrian guidelines also include legume-based products such as tofu and tempeh, which is consistent with the recommendations of the Planetary Health Diet.

#### 4.1.2 Milk and dairy

Milk and dairy products are important sources of calcium, Vitamin B_12_, and iodine (18), but they have relatively high GHGE (6). Milk and dairy recommendations are broadly consistent across the examined dietary guidelines, with only minor variations. The updated Austrian guidelines recommend two servings of milk and dairy products per day, aligning with actual consumption trends in Austria (15, 37, 53). This is in line with the German guidelines (14). The NNRs suggest a daily intake of 350–500 mL, which is comparable in quantity (10). In the Netherlands, the recommendation varies by target group, ranging from two to four servings per day (9). In contrast, the Planetary Health Diet proposes a more conservative intake of 250 g/day, with a flexible range of 0–500 g/day (7). The UK’s Eatwell Guide includes dairy within its protein-rich food group but does not specify a quantitative recommendation (44). Regarding plant-based alternatives, all guidelines suggest the use of fortified plant-based drinks when dairy intake is below the recommended amounts to ensure an adequate intake of key nutrients such as calcium, Vitamin B_12_, and Vitamin B_2_ (10, 43, 44, 54). To reflect changing consumption habits, plant-based milk alternatives were included in the Austrian optimization process. Sales of these drinks rose by 21% in Austria between 2020 and 2022 (55). However, they are not nutritionally equivalent to cow’s milk and should be fortified with calcium, Vitamin B_12_, and B_2_ if used as substitutes. Environmentally, plant-based drinks have a lower impact than cow’s milk, though this varies by product and production method (6, 56).

#### 4.1.3 Eggs

Of the dietary guidelines compared, Austria’s are the most permissive with regard to egg intake, allowing up to three eggs per week, which corresponds to approximately 26 g/day. The Netherlands position themselves in the mid-range with a recommendation of two to three eggs per week (9). In contrast, Germany and the Planetary Health Diet advocate for more limited consumption, recommending one egg per week and approximately 1.5 eggs per week, respectively (7, 14). The UK’s Eatwell Guide includes eggs as part of a balanced protein intake but does not provide a specific quantitative recommendation (44).

#### 4.1.4 Fish

Fish consumption is encouraged across all examined dietary guidelines due to its well-established health benefits (57) and its role as a valuable source of long-chain omega-3 fatty acids, iodine, and Vitamin B12 (18). Most guidelines specifically emphasize the importance of fatty fish, given its higher omega-3 content. The Austrian FBDGs recommend approximately 29 g/day, equivalent to 1–2 servings per week, a recommendation shared by Germany (58). The Planetary Health Diet similarly suggests 28 g/day (range: 0–100 g/day) (7). The UK’s Eatwell Guide advises two portions per week, corresponding to roughly 40 g/day (44). The NNRs and Norwegian guidelines propose the highest intake, recommending 300–450 g/week (≈43–64 g/day) (10, 43). This reflects both regional dietary patterns and the cultural significance of fish in Nordic countries, where consumption is traditionally higher than in Austria (59). However, the NNRs, like Austria, also stress the importance of sourcing fish from sustainably managed farms and wild stocks and advise limiting species with high environmental impact. In contrast, the Netherlands recommend the lowest intake, with 14 g/day (9).

#### 4.1.5 Meat

A consistent topic across all dietary guidelines is the recommendation to reduce meat consumption, particularly red and processed meat, due to its adverse health effects and environmental impact (36, 60–62). In alignment with the Planetary Health Diet, Austria and Germany recommend a maximum intake of 43 g/day (≈300 g/week) (7, 14, 37). In comparison to the previous Austrian FBDGs, the recommended weekly meat intake has been reduced from 300 to 450 g per week to a maximum of 300 g of meat per week. For Austria, the reduction is supported by the modeling results demonstrating significant reductions in GHGE and LU when compared to current Austrian consumption patterns. The NNRs and recommendations of Norway are quite similar, at 350 g/week (10, 43). The Netherlands and the UK adopt a more moderate stance, advising a maximum intake of around 70 g/day (9, 44). Importantly, the NNRs state that reducing red meat intake should not lead to increased consumption of white meat, but rather to an increased consumption of, e.g., legumes and fish. Additionally, the intake of processed white meat is advised to be kept as low as possible (10).

### 4.2 Strengths and limitations

Our study presents several strengths. First, all nutrient reference values were considered for mathematical modeling. Second, beyond evaluating nutritional adequacy, the analysis incorporated recent and robust data on DALYs to estimate the potential health impacts of the modeled diets. Third, cultural acceptability was taken into account to ensure the practical feasibility of the proposed dietary patterns. Lastly, the inclusion of plant-based meat and dairy alternatives represents a significant strength, as it aligns with current consumer behavior and enhances the relevance of the dietary scenarios.

However, our study presents also some limitations. First, in this study, GHGE and LU were selected as the primary environmental indicators due to the availability of robust and reliable data (63). In the future, it would be interesting to also include other environmental parameters such as water use or marine eutrophication. However, GHGE is currently the most frequently applied indicator (64) and shows strong correlations with other environmental parameters (65). Second, due to limitations of national data we had to use European data on environmental footprints. However, in order to accurately reflect Austrian agricultural conditions, slight manual adjustments were made in the analysis of the results. Third, some nutrients (such as selenium, iodine, manganese, copper, and fluoride) had to be excluded due to limited data quality in the nutrient databases used. However, iodized salt has been used in Austria since 1963 to improve population’s iodine status (66) and remains a key strategy for ensuring adequate intake. Additionally, owing to iodine-fortified animal feed (67), milk, dairy products, and eggs represent important dietary sources of iodine and are included in the omnivorous FBDGs. Also, animal feed may be fortified with selenium in the European Union (68). As a result, animal-derived foods such as meat and eggs can contribute relatively consistently to selenium intake.

A key strength of the applied modeling framework is its adaptability: it can be readily updated as more refined data on nutrient composition, sustainability metrics, or dietary intake data of different target groups become available, thereby supporting the continuous improvement of FBDGs.

### 4.3 Conclusion

The newly developed FBDGs represent a significant advancement in integrating nutritional adequacy and health aspects with environmental sustainability for Austria. In summary, the updated Austrian FBDGs provide clear, quantitative recommendations that ensure nutritional adequacy, suggest a potential reduction in DALYs by 43%, and could achieve a reduction in GHGE by 58% and LU by 61%, compared to the observed diet of the Austrian population. Our study supports previous findings (9, 12, 69, 70) that mathematical optimization is a robust and integrative approach for developing FBDGs, as it enables the simultaneous consideration of multiple dimensions of health and sustainability. We showed that a critical analysis of modeling results and manual adjustments, if required, are necessary steps to achieve realistic, practical outcomes tailored to the specific characteristics of a country. The updated FBDGs are high in vegetables, fruits, grains and legumes and contain only small amounts of animal-based foods. The FBDGs provide guidance on the path to a healthy and environmentally friendly diet. As current meat consumption in Austria far exceeds the recommendations, gradual dietary shifts are required. An increased intake of legumes and minimally processed legume products can facilitate the shift to more sustainable diets. To achieve this, it is necessary to disseminate the updated FBDGs widely to the general public and to experts through communication measures.

## Data Availability

We did not generate data in the context of this article that are not shown in the tables and supplements. The main data sources were from third parties that can easily be contacted via the information given in the text and references. MS-Nutrition has provided the basic programming (contact at: https://ms-nutrition.com/en/).
