# Supplementary material for: Reducing disease burden through sustainable diets: a modeling approach for national food-based dietary guidelines
Source: Front Nutr. 2026 Jul 17;13:1826425. doi: 10.3389/fnut.2026.1826425 (PMC13423645; doi:10.3389/fnut.2026.1826425)
Supplement: Supplementary file 3 [file Table_3.DOCX]

**Supplement 3: Overview of the nutrient goals**

| Nutrient | Type | Lower level | Upper level |
| --- | --- | --- | --- |
| Fat, E% | GV | - | 30 |
| Saturated fatty acids, E% | n.a. | - | 10 |
| Monounsaturated fatty acids, E% | n.a. | 10 | - |
| Polyunsaturated fatty acids, E% | n.a. | 7 | 10 |
| Linoleic acid, E% | RI | 2.5 | - |
| α-Linolenic acid, E% | EST | 0.5 | - |
| Docosahexaenoic acid | EST | 250* |  |
| Cholesterol (mg/d) | GV | - | 300 |
| Protein (g/d) | RI | 52 | - |
| Carbohydrates, E% | n.a. | - | - |
| Free sugars, E% | n.a. | - | 10** |
| Fiber (g/d) | GV | 30 | - |
| Alcohol (g/d) | GV | - | 10*** |
| Vitamin A (Retinol Activity Equivalent µg/d) | RI | 776 | - |
| Vitamin E (Equivalent mg/d) | EST | 13 | 300**** |
| Vitamin K1 (µg/d) | EST | 68 | - |
| Thiamin (mg/d) | RI | 1.1 | - |
| Riboflavin (mg/d) | RI | 1.2 | - |
| Niacin (Equivalent mg/d) | RI | 13.4 | - |
| Pantothenic acid (mg/d) | EST | 5 | - |
| Vitamin B6 (mg/d) | RI | 1.5 | 25**** |
| Biotin (µg/d) | EST | 40 | - |
| Folate (µg/d) | RI | 300 | 1000**** |
| Vitamin B12 (µg/d) | EST | 4 | - |
| Vitamin C (mg/d) | RI | 103 | - |
| Sodium (mg/d) | EST | 1500 | 2400**** |
| Chloride (mg/d) | EST | 2300 | - |
| Potassium (mg/d) | EST | 4000 | - |
| Calcium (mg/d) | RI | 1000 | 2500**** |
| Phosphorus (mg/d) | RI | 700 | - |
| Magnesium (mg/d) | EST | 325 | - |
| Iron (mg/d) | RI | 15*** | - |
| Iodine (µg/d) | RI | -***** | 600***** |
| Fluoride (mg/d) | GV | - | 7***** |
| Zinc (mg/d) | RI | 11 | 25**** |
| Copper (mg/d) | EST | - | 5***** |
| Water (ml/d) | GV | 2161 | - |
|  |  | **Equivalent** | |
| Energy, kcal | GV | 2029 |  |

E%: Percentage of daily energy intake, GV = guiding value, RI = recommended intake, EST = estimated value for an adequate intake,

DRVs from DGE/ÖGE (1) unless differently indicated

* European Food Safety Authority (2)

**Recommendation of the World Health Organization (3)

*** Reference value for women instead of weighted average

****European Food Safety Authority Dietary Reference Value/Upper Intake Level (4)

*********Exclusion due to data weakness

1. Deutsche Gesellschaft für Ernährung e.V., Österreichische Gesellschaft für Ernährung. *Referenzwerte Für Die Nährstoffzufuhr*. 2 ed. Bonn: Deutsche Gesellschaft für Ernährung und Österreichische Gesellschaft für Ernährung, (2024).

2. European Food Safety Authority. Scientific Opinion on the Tolerable Upper Intake Level of Eicosapentaenoic Acid (Epa), Docosahexaenoic Acid (Dha) and Docosapentaenoic Acid (Dpa). *EFSA Journal* (2012) 10(48). doi: <https://doi.org/10.2903/j.efsa.2012.2815>

3. World Health Organization. Guideline: Sugars Intake for Adults and Children. Geneva: World Health Organization (2015). Available from: <https://iris.who.int/bitstream/handle/10665/149782/9789241549028_eng.pdf?sequence=1>

4. European Food Safety Authority. Overview on Tolerable Upper Intake Levels as Derived by the Scientific Committee on Food and the Efsa Panel on Dietetic Products, Nutrition and Allergies. Parma: EFSA (2024). Available from: <https://www.efsa.europa.eu/sites/default/files/2024-05/ul-summary-report.pdf>
