# Supplementary material for: Reducing disease burden through sustainable diets: a modeling approach for national food-based dietary guidelines
Source: Front Nutr. 2026 Jul 17;13:1826425. doi: 10.3389/fnut.2026.1826425 (PMC13423645; doi:10.3389/fnut.2026.1826425)
Supplement: Supplementary file 4 [file Table_4.DOCX]

**Supplement 4: Dietary patterns of the different scenarios**

| Food groups and subgroups | Observed diet (g/d) (1) | Scenario 1 (g/d) | Scenario 2 (300g meat per week) (g/d) | Scenario 3 (2 portions of fish per week) | Scenario 4 (1,5 portion of meat per week) (g/d) | Adapted scenario 4, adapted for FBDG (g/d) |
| --- | --- | --- | --- | --- | --- | --- |
| Drinking water | 1626 | 1061 | 1044 | 1025 | 1039 | 1039 |
| Coffee and tea | 532 | 126 | 126 | 126 | 126 | 126 |
| Vegetables and fruit | 394 | 670 | 669 | 653 | 672 | 672 |
| Vegetables | 183 | 329 | 328 | 314 | 331 | 331 |
| Fruit | 131 | 261 | 261 | 259 | 261 | 261 |
| Fruit and vegetable juices | 80 | 80 | 80 | 80 | 80 | 80 |
| Grains, grain-based products and potatoes | 301 | 378 | 366 | 414 | 378 | 378 |
| Refined grains (-products) | 229 | 255 | 246 | 231 | 247 | 247 |
| Whole grains (-products) | 17 | 17 | 17 | 17 | 17 | 17 |
| Potatoes | 55 | 106 | 103 | 166 | 114 | 114 |
| Legumes and legume products | 13 | 57 | 40 | 34 | 37 | 37 |
| Legumes (cooked) | 12 | 12 | 12 | 12 | 13 | 13 |
| Plant-based meat substitutes made of legumes | 1 | 45 | 28 | 22 | 24 | 24 |
| Eggs | 23 | 13 | 13 | 48 | 13 | 26 |
| Milk equivalents** | 571 | 326 | 346 | 350 | 346 | 411 |
| Milk/dairy (in milk equivalents) | 561 | 297 | 301 | 298 | 296 | 361 |
| Plant-based drinks | 10 | 29 | 45 | 52 | 50 | 50 |
| Fats, oils, nuts and seeds | 25 | 25 | 25 | 24 | 26 | 26 |
| Vegetable oils | 8 | 9 | 9 | 8 | 10 | 10 |
| Nuts and seeds | 8 | 8 | 8 | 8 | 8 | 8 |
| Spreadable fats | 9 | 8 | 8 | 8 | 8 | 8 |
| Meat | 128 | 11 | 43 | 11 | 32 | 32 |
| Red meat | 56 | 6 | 6 | 6 | 6 | 6 |
| Processed meat | 44 | 5 | 5 | 5 | 5 | 5 |
| Poultry | 28 | 0 | 31 | 0 | 21 | 21 |
| Fish and seafood | 19 | 52 | 46 | 38 | 48 | 29 |
| Discretionary foods | 21 E% | 10 E% | 10 E% | 9 E% | 10 E% | 10 E% |

1. Rust P, Hasenegger V, König J. Österreichischer Ernährungsbericht 2017. Wien: Bundesministerium für Gesundheit und Frauen (2017). Available from: <https://broschuerenservice.sozialministerium.at/Home/Download?publicationId=528>
